# A novel AMPK activator hernandezine inhibits LPS-induced TNFα production

**DOI:** 10.18632/oncotarget.18365

**Published:** 2017-06-05

**Authors:** Ping Li, Xiaofang Li, Yonghong Wu, Manxiang Li, Xiaochuang Wang

**Affiliations:** ^1^ Department of Emergency, The Second Affiliated Hospital of Xi’an Jiao Tong University, Xi’an, China; ^2^ Department of Gastroenterology, The Third People’s Hospital of Xi’an, Xi’an, China; ^3^ Staff Room of Clinical Immunology and Pathogen Detection, Medical Technology Department, Xi’an Medical College, Xi’an, China; ^4^ Department of Respiratory Medicine, The First Affiliated Hospital of Xi’an Jiao Tong University, Xi’an, China; ^5^ Department of Critical Care Medicine, The Second Affiliated Hospital of Xi’an Jiao Tong University, Xi’an, China

**Keywords:** hernandezine, LPS, AMPK, TNFα, NFκB

## Abstract

Here, we found that hernandezine, a novel AMPK activator, inhibited LPS-induced TNFα expression/production in human macrophage cells (THP-1 and U937 lines). Activation of AMPK is required for hernandezine-induced anti-LPS response. AMPKα shRNA or dominant negative mutation (T172A) blocked hernandezine-induced AMPK activation, which almost completely reversed anti-LPS activity by hernandezine. Exogenous expression of the constitutively activate AMPKα (T172D, caAMPKα) also suppressed TNFα production by LPS. Remarkably, hernandezine was unable to further inhibit LPS-mediated TNFα production in caAMPKα-expressing cells. Hernandezine inhibited LPS-induced reactive oxygen species (ROS) production and nuclear factor kappa B (NFκB) activation. Treatment of hernandezine in *ex-vivo* cultured primary human peripheral blood mononuclear cells (PBMCs) also largely attenuated LPS-induced TNFα production. Together, we conclude that AMPK activation by hernandezine inhibits LPS-induced TNFα production in macrophages/monocytes.

## INTRODUCTION

Patients with chronic obstructive pulmonary disease (COPD) often suffer chronic yet persistent airway inflammations [1–3]. Many pathogen-associated molecular patterns (PAMPs) are circulating in lungs of the COPD patients [1–3]. Lipopolysaccharide (LPS) is one of the most prominent PAMPs [4, 5]. LPS activates resident monocytes/macrophages to produce TNFα (tumor necrosis factor-α) and other pro-inflammatory cytokines [4, 5]. TNFα level is significantly elevated in bronchoalveolar lavage fluids, sputum, as well as plasma and lung of COPD patients [6–8]. Anti-TNFα strategy could efficiently lessen COPD patients’ inflammations [6–8]. The research focus of our group is to explore the underlying mechanisms of LPS-induced TNFα production, which could possibly help to develop intervention agents [9–11].

AMP-activate protein kinase (AMPK) is the key sensor of cellular energy status [12, 13]. Evidences (including ours [9–11]) have implied a key function of AMPK in suppressing inflammatory responses [14–17]. Several AMPK activators, including AICAR, A769662 and GSK621, significantly attenuated LPS-mediated nuclear factor kappa B (NFκB) activation and cytokine production [10, 14, 15]. Metformin, another AMPK activator, attenuated expression of pro-inflammatory and adhesion molecule [18]. Further, perifosine activated AMPK signaling and inhibited LPS-induced TNFα production [15]. Cordycepin-activated AMPK also significantly inhibited TNFα expression by LPS [19]. Thus, AMPK activation represents a novel and efficient strategy to inhibit LPS inflammatory response [10, 11, 14, 15, 18, 19].

Hernandezine is an alkaloid isolated from Chinese medicinal herb *manyleaf meadowure rhizome and root* [20]. A very recent study by Law *et al.*, has characterized hernandezine as a novel AMPK activator [20]. In the current report, we show that hernandezine inhibits LPS-induced TNFα production via activating AMPK signaling.

## RESULTS

### The effect of hernandezine on macrophage cell survival and TNFα production

**Figure 1:**
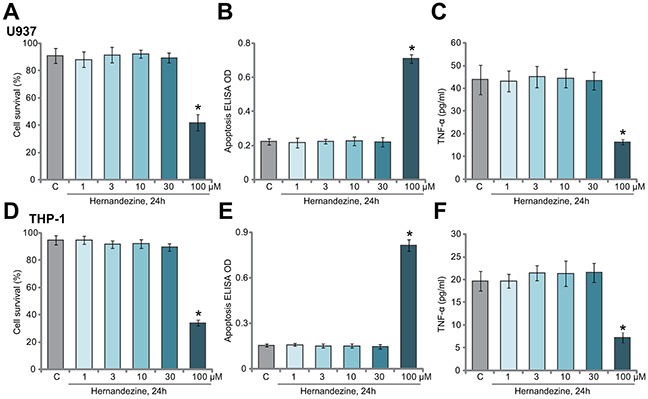
The effect of hernandezine on macrophage cell survival and TNFα production Human macrophage cells, U937 line **(A-C)** and THP-1 line **(D-E)**, were either left untreated (“C”, same for all figures) or treated with hernandezine (1-100 μM), cells were further cultured for 24 hours, cell survival (**A and D**, trypan blue assay), apoptosis (**B and E**, Histone DNA ELISA assay) and medium TNFα content (**C and F**, ELISA assay) were tested. *p<0.05 *vs.* “C”. Experiments in this figure were repeated three times, and similar results were obtained.

First, we tested the potential effect of hernandezine on the survival of human macrophage cells. U937 cells (macrophage cell line [11]) were treated with gradually increased concentrations of hernandezine (1-100 μM) for 24 hours, trypan blue staining assay [11] was applied to test cell survival. Viable cells were trypan blue negative [11]. Results in Figure 1A demonstrated that hernandezine was not cytotoxic to U937 cells until at 100 μM, the latter induced obvious U937 cell death (Figure 1A). Histone DNA apoptosis ELISA assay [10, 11] results in Figure 1B demonstrated that only 100 μM of hernandezine induced significant U937 cell apoptosis. It was not pro-apoptotic at lower concentrations (Figure 1B). As shown in Figure 1C, treatment with hernandezine (1-30 μM, non-cytotoxic concentrations) failed to change basal TNFα production in U937 cells. However, at 100 μM, hernandezine inhibited TNFα production (Figure 1C), which could be probably due to cell death (Figure 1A and 1B). The similar experiments were also performed in the other human macrophage cell line: THP-1 [11]. Results showed that hernandezine was indeed not cytotoxic (Figure 1D) nor pro-apoptotic (Figure 1E) to THP-1 cells until at a high concentration (100 μM). Basal TNFα production in THP-1 cells was also not changed in hernandezine-treated THP-1 cells, except at 100 μM (Figure 1F).

### The effect of hernandezine on LPS-induced TNFα production in macrophage cells

**Figure 2:**
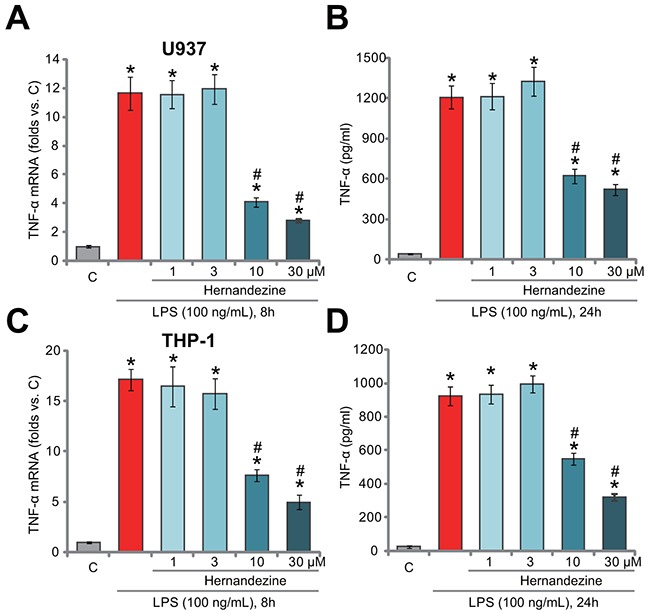
The effect of hernandezine on LPS-induced TNFα production in macrophage cells Human macrophage cells, U937 line **(A and B)** and THP-1 line **(C and D)**, were treated with LPS (100 ng/mL) or plus hernandezine (1-30 μM), cells were further cultured in the conditional medium for indicated time, TNFα mRNA expression **(A and C)** and TNFα protein content (in conditional medium, **B and D**) were tested by qRT-PCR assay and ELISA assay, respectively. *p<0.05 *vs.* “C”. ^#^ *p*<0.05 *vs.* LPS only treatment. Experiments in this figure were repeated three times, and similar results were obtained.

One main focus of this study is to test the potential effect of hernandezine on LPS-induced pro-inflammatory activity. In line with our previous findings [11], treatment with LPS (100 ng/mL) induced dramatic TNFα mRNA expression (Figure 2A) and protein secretion (Figure 2B) in U937 cells. Remarkably, co-treatment with hernandezine (at 10 and 30 μM), significantly attenuated LPS-induced TNFα expression and production (Figure 2A and 2B). Hernandezine demonstrated a dose-dependent response in inhibiting TNFα production (Figure 2A and 2B). At lower concentrations (1 and 3 μM), hernandezine was in-effective on LPS (Figure 2A and 2B). Notably, similar results were also obtained in THP-1 macrophage cells, where hernandezine (10/30 μM) largely attenuated LPS (100 ng/mL)-induced TNFα mRNA expression (Figure 2C) and production (Figure 2D). Once again, hernandezine at 1/3 μM was ineffective in THP-1 cells (Figure 2C and 2D). These results demonstrate that hernandezine inhibits LPS-induced TNFα production in macrophage cells.

### Activation of AMPK is required for hernandezine-induced anti-LPS response

**Figure 3:**
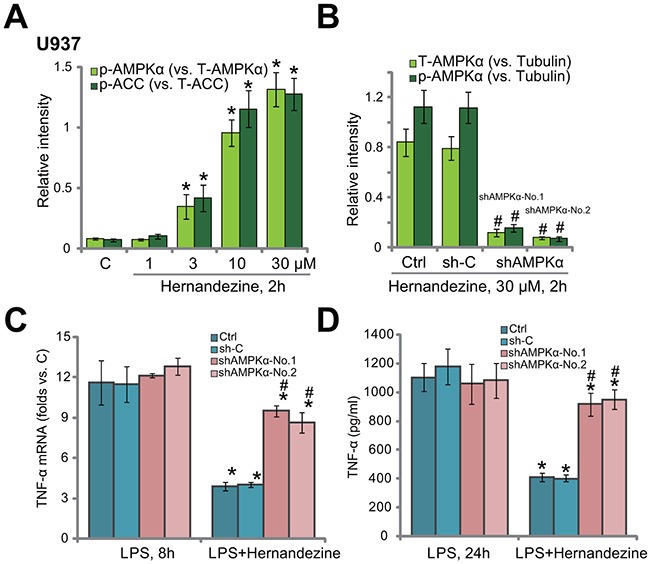
Activation of AMPK is required for hernandezine-induced anti-LPS response U937 cells were treated with hernandezine (1-30 μM) for 2 hours, expression of listed proteins was tested by Western blotting assay, and blot data of three sets of repeat were quantified **(A)** U937 cells were infected with lentiviral AMPKα shRNA (“shAMPKα-No.1”/“shAMPKα-No.2”) or scramble control shRNA (“sh-C”), and stable cells were established; cells were treated with hernandezine (30 μM) for 2 hours, expression of listed proteins was tested by Western blotting assay (blot data of three sets of repeat were quantified **(B)**); cells were also treated with LPS (100 ng/mL), TNFα mRNA expression **(C)** and protein content (in conditional medium, **(D)**) were tested. “Ctrl” stands for un-infected cells. *p<0.05 *vs.* “C” **(A)**. ^#^ *p*<0.05 *vs.* “sh-C” group **(B)**. *p<0.05 *vs.* LPS only group **(C and D)**. ^#^ *p*<0.05 *vs.* hernandezine of “sh-C” group **(C and D)**. Experiments in this figure were repeated three times, and similar results were obtained.

Our group [10, 11] and others [15, 16, 19] have implied that activation of AMPK could inhibit LPS-induced pro-inflammatory response. The study by Law *et al.*, has confirmed that hernandezine is a novel AMPK activator [20]. We therefore tested AMPK signaling in hernandezine-treated macrophage cells. As shown in Figure 3A, hernandezine dose-dependently induced AMPK activation in U937 cells. As phosphorylated- (“p-”) AMPKα and p-acetyl-CoA carboxylase (p-ACC, the main downstream target protein of AMPK [12, 21]) were significantly increased after treatment of 10 and 30 μM of hernandezine (See quantified blot results in Figure 3A). On the other hand, 1 μM and 3 μM of hernandezine failed to induce significant AMPK activation (See quantified blot results in Figure 3A). Total AMPKα and ACC expression was unchanged following hernandezine treatment.

To study the link between AMPK activation and hernandezine-induced anti-LPS response, shRNA method was utilized to knockdown AMPKα. In line with our previous studies [10, 11], two AMPKα shRNAs with non-overlapping sequences were applied. The two shRNAs were named as “shAMPKα-No.1” and “shAMPKα-No.2” [10, 11]. AMPKα was indeed silenced in U937 cells expressing the AMPKα shRNA (See quantified blot results in Figure 3B). Consequently, hernandezine-induced AMPK activation, or p-AMPKα, was almost blocked (See quantified blot results in Figure 3B). AMPKα shRNAs didn’t change LPS-induced TNFα mRNA expression (Figure 3C) and production (Figure 3D) in U937 cells. Remarkably, in AMPKα-silenced U937 cells, hernandezine-induced anti-LPS response was largely compromised (Figure 3C and 3D). In another words, hernandezine was unable to inhibit LPS-induced TNFα synthesis (Figure 3C) and production (Figure 3D) when AMPK was silenced. These results imply that activation of AMPK is required for hernandezine-induced anti-LPS response.

### AMPKα dominant negative mutation abolishes hernandezine-induced anti-LPS response

**Figure 4:**
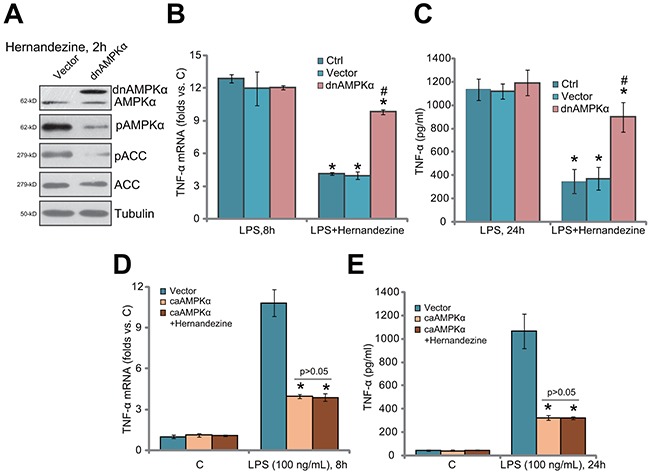
AMPKα dominant negative mutation abolishes hernandezine-induced anti-LPS response U937 cells were constructed with the dominant negative mutant AMPKα (T172A, “dnAMPKα”), the constitutively activate AMPKα (T172D, “caAMPKα”) or the empty vector (pSuper-puro, “Vec”), and stable cells were established; Cells were treated with hernandezine (30 μM) or plus LPS (100 ng/mL) for indicated time, expression of listed proteins was tested by Western blotting assay **(A)**; TNFα mRNA expression **(B and D)** and protein content (in conditional medium, **(C and E)** were tested by qRT-PCR assay and ELISA assay, respectively. *p<0.05 *vs.* LPS only group. ^#^ *p*<0.05 *vs.* hernandezine of “Vec” group. Experiments in this figure were repeated three times, and similar results were obtained.

To further support the requirement of AMPK activation in hernandezine-induced anti-LPS response, a dominant negative mutant AMPKα (T172A, “dnAMPKα”) [11, 22–24] was introduced to U937 cells. Western blotting assay results in Figure 4A confirmed the expression of the dnAMPKα in the stable U937 cells. Notably, hernandezine-induced AMPK activation, or p-AMPKα/ACC, was almost blocked in dnAMPKα-expression U937 cells (Figure 4A). Consequently, hernandezine-induced anti-LPS response was significantly attenuated (Figure 4B and 4C). Hernandezine was largely ineffective against LPS-induced TNFα mRNA expression (Figure 4B) and production (Figure 4C) when AMPK was mutant. Next, the constitutively-activate AMPKα (T172D, “caAMPKα”) [10, 23] was introduced to U937 cells. Stable cells with caAMPKα were again established. As demonstrated, LPS-induced TNFα mRNA expression (Figure 4D) and production (Figure 4E) were largely attenuated in the caAMPKα-expressing U937 cells. Remarkably, the anti-LPS activity of hernandezine was nullified in caAMPKα-expressing cells (Figure 4D and 4E). Hernandezine was unable to further suppress LPS-induced TNFα production when AMPK was already constitutively-activated (Figure 4D and 4E). These results again confirmed that activation of AMPK is required for hernandezine-induced anti-LPS activity.

### Hernandezine inhibits LPS-induced ROS production and NF-kB activation

**Figure 5:**
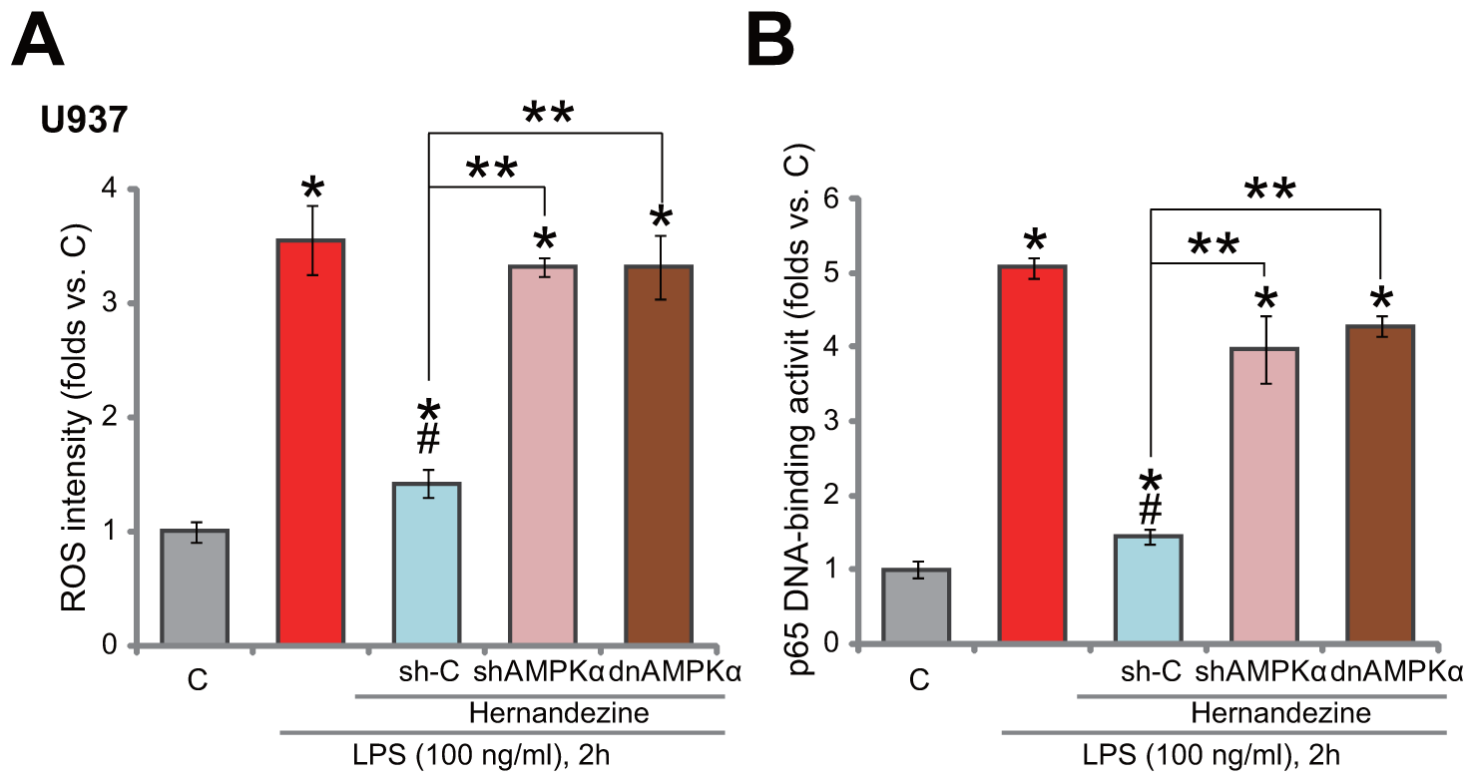
Hernandezine inhibits LPS-induced ROS production and NF-kB activation Stable U937 cells, with scramble control shRNA (“sh-C”), AMPKα shRNA (“shAMPKα”, No.1) or dominant negative AMPKα (T172A, “dnAMPKα”), were treated with LPS (100 ng/mL) or plus hernandezine (30 μM) for indicated time, relative ROS intensity **(A)** and NFκB activation **(B)** were tested. * *p* < 0.05 v*s.* “C” group.^#^ *p* < 0.05 *vs.* LPS only group. ** *p* < 0.05. Experiments in this figure were repeated three times, and similar results were obtained.

As discussed, forced-activation of AMPK was shown to efficiently suppress LPS-induced ROS production and subsequent nuclear factor kB (NF-kB) activation [10, 11, 15, 16, 19], leading to TNFα transcription inhibition in monocytes/macrophages. Here, we showed that treatment with hernandezine (30 μM) in U937 cells largely attenuated LPS-induced ROS production (Figure 5A). Further, NF-kB activation in LPS-treated U937 cells was also inhibited by hernandezine (Figure 5B). Importantly, AMPKα knockdown (by targeted shRNA, shAMPKα-No.1) or mutation (by expressing dnAMPKα) almost abolished hernandezine-induced inhibition on ROS and NF-kB (Figure 5A and 5B). These results suggest that AMPK activation is required for hernandezine-induced inhibition on ROS production and NF-kB activation in LPS-treated cells.

### Hernandezine inhibits LPS-induced TNFα production in primary human peripheral blood mononuclear cells (PBMCs)

**Figure 6:**
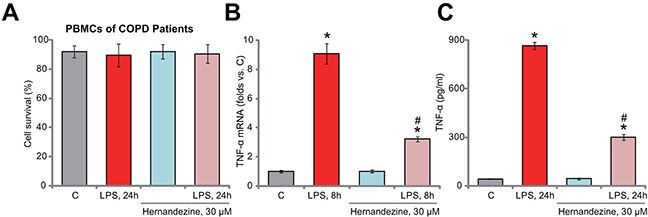
Hernandezine inhibits LPS-induced TNFα production in primary human PBMCs *Ex-vivo* cultured PBMCs of COPD patients were treated with LPS (100 ng/mL) and/or hernandezine (30 μM) for indicated time, cell survival was tested by trypan blue assay **(A)**; relative TNFα mRNA expression (**(B)**, qRT-PCR assay) and TNFα content in conditional medium (**(C)**, ELISA assay) were also tested. * *p* < 0.05 *vs.* “C” group. ^#^ *p* < 0.05 *vs.* LPS only group. Experiments in this figure were repeated three times, and similar results were obtained.

At last, we tested the potential activity of hernandezine in human monocytes. In consistent with our previous studies [9, 10], primary PBMCs from COPD patients were *ex-vivo* cultured. Trypan blue assay results in Figure 6A showed again that treatment with 30 μM of hernandezine (or plus LPS) was non-cytotoxic to the primary PBMCs. Significantly, hernandezine remarkably inhibited LPS-induced TNFα mRNA expression (Figure 6B) and production (Figure 6C). Therefore, in line with the cell line data, hernandezine similarly inhibits LPS-induced TNFα production and expression in primary human PBMCs.

## DISCUSSION

We have previously shown that GSK621, the novel AMPK activator [25], attenuated LPS-induced TNFα production [10]. Similarly, our very recent study has demonstrated that microRNA-135b-5p (“miR-135b-5p”) inhibited LPS-induced TNFα production via activating AMPK [11]. miR-135b-5p activated AMPK signaling via silencing its phosphatase Ppm1e [11]. These results indicate that AMPK activation could be a novel and efficient strategy to inhibit LPS-induced pro-inflammatory response. Here, we showed that AMPK activation is also required for hernandezine-mediated anti-LPS response. AMPK knockdown (by targeted shRNAs) or dominant negative mutation blocked hernandezine-induced AMPK activation, and almost completely reversed its anti-LPS activity. Meanwhile, exogenous expression of caAMPKα also inhibited LPS-induced TNFα production. Importantly, hernandezine was almost invalid against LPS in the caAMPKα-expressing cells. Thus, activation of AMPK by hernandezine is responsible for its anti-LPS activity in macrophage cells.

LPS, which is sensed by CD14 and LPS-binding protein, binds to Toll-like receptor 4 (TLR-4) on macrophages/monocytes [26, 27], which will activate downstream NFκB signaling to initiate pro-inflammatory response [26, 27]. ROS production is known to be critical in the process. Recent studies [19, 28, 29] including ours [11] have implied that AMPK could be effective in suppressing oxidative stresses. For example, energy depletion-activated AMPK-ACC signaling was shown to increase intracellular nicotinamide adenine dinucleotide phosphate (NADPH) content and to inhibit oxidative stress [28]. She *et al.,* demonstrated that activation of AMPK significantly suppressed H_2_O_2_-induced oxidative damages [29]. Similarly, AMPK activation by cordycepin inhibited LPS-induced ROS accumulation [19]. Our previous studies have shown that GSK621 [10] or miR-135b-5p [11] inhibited LPS-induced ROS production, thus blocking the downstream NFκB activation. This could be the key mechanism responsible for AMPK-induced anti-LPS activity.

In line with these findings, we show that hernandezine largely inhibited LPS-induced ROS production and NFκB activation in U937 cells. AMPK inhibition, by targeted shRNA or dominant negative mutation, almost completely reversed hernandezine’s above actions. Thus, we conclude that hernandezine activates AMPK signaling to inhibit LPS-induced ROS production and subsequent NFκB activation, which then leads to decreased TNFα mRNA synthesis and production. The detailed mechanism may warrant further investigations.

## MATERIALS AND METHODS

### Chemicals and antibodies

Hernandezine was purchased from EFE-Bio Company (Shanghai, China). LPS and puromycin were provided from Sigma Chemicals (Shanghai, China). The antibodies were all obtained from Cell Signaling Technology (Danvers, MA). Cell culture reagents were provided by Hyclone (Shanghai, China).

### Cell culture

As described previously [11], the two human macrophage cell lines, U937 and THP-1, were cultured in RPMI 1640 medium supplemented with 10% FBS and 1% glutamine at 37°C.

### *Ex-vivo* culture of human PBMCs

As described previously [9, 10], PBMCs of COPD patients (administrated at the Second Affiliated Hospital of Xi’an Jiao Tong University, Xi’an, China) were collected via lymphocyte separation medium (Sigma, Shanghai, China). The resulting PBMCs were cultured in DMEM plus 10% FBS, and necessary supplements [30]. Experiments and protocols requiring human samples were approved by the Ethics Committee and Internal Review Board of Xi’an Jiao Tong University. Written-informed consent was provided by each patient.

### Real-time PCR assay

The detailed protocol for real-time reverse transcriptase quantitative polymerase chain reaction (qRT-PCR) assay was described in previous studies [9–11]. The primers for TNFα mRNA and GAPDH mRNA were described previously [11]. All primers were synthesized by Genepharm (Shanghai, China). We utilized the comparative Ct (2^−ΔΔCt^) method to calculate relative *TNFα mRNA expression* [31–33]. *GAPDH was always tested as the reference gene* [33].

### TNFα enzyme-linked immunosorbent assay (ELISA) assay

TNFα protein content in the conditional medium was tested by the TNFα ELISA kit (R&D Systems, Abingdon, UK), and detailed protocol was described previously [9].

### Western blotting assay

As described [9–11], after applied treatment, cells were lysed, the protein lysates (20 μg per sample) were separated by the SDS-PAGE gel (10-12%). Protein samples were then transferred onto PVDF membranes, which were then probed with indicated primary and corresponding secondary antibodies. The indicated bands were then visualized by the enhanced chemiluminescence (ECL, Amersham, Shanghai, China) regents [9].

### AMPKα shRNA

The two lentiviral human AMPKα short hairpin RNAs (shRNAs, “No1” and “No2”, with non-overlapping sequences) were described previously [10, 11, 34]. Cells were incubated with the lentiviral shRNA for 24 hours, and were selected by puromycin (1.0 μg/mL) for another 12 days [10, 11, 34]. Western blotting assay was applied to confirm the stable knockdown of AMPKα. The non-sense lentiviral control shRNA (Santa Cruz Biotech) was added to the control cells.

### AMPKα mutation

The pSuper-puro construct with dominant negative AMPKα (T172A), the constitutively-active AMPKα (T172D), and the empty vector were provided by Dr. Lu’s group [10, 11, 34]. We utilized Lipofectamine 2000 to transfect the mutant AMPKα or the empty vector to U937 cells. Stable cells were again selected by puromycin.

### ROS assay

The detailed protocol for ROS assay was described previously [10, 11, 19]. Briefly, ROS content in cells with applied treatment was measured by dichlorofluorescin (DCF) oxidation assay. Cells were incubated with 10 μM of DCFH-DA (Invitrogen, Shanghai, China) for 30 min, and were then washed in PBS for three times. DCF fluorescence intensity was then tested [10, 11, 19].

### Measuring NFκB (p65) DNA-binding activity

NFκB (p65) DNA-binding activity was tested as described in our previous studies [9–11]. Briefly, after applied treatment, 1.0 μg of cell nuclear extracts per treatment were analyzed of the NFκB (p65) DNA-binding activity, via the TransAM™ ELISA kit (Active Motif, Carlsbad, CA) according to the recommended protocol. The OD value of treatment group was normalized to that of control group to reflect relative NFκB activity.

### Statistics analysis

The statistical analyses were performed via the SPSS software (18.0), with *p* < 0.05 taken as significant. Data were expressed as mean ± standard deviation (SD). For comparisons among multiple groups, two-way ANOVA with the Bonferroni post hoc testing was performed.
